# Crystal structure of 10-benzyl-9-(3,4-di­meth­oxy­phen­yl)-3,3,6,6-tetra­methyl-3,4,6,7,9,10-hexa­hydro­acridine-1,8(2*H*,5*H*)-dione

**DOI:** 10.1107/S2056989015014966

**Published:** 2015-08-26

**Authors:** N. Sureshbabu, V. Sughanya

**Affiliations:** a Department of Chemistry, Annamalai University, Annamalai Nagar 608 002, Tamil Nadu, India

**Keywords:** crystal structure, dimedone, benzyl­amine, acridinedione

## Abstract

In the acridinedione moiety of the title compound, C_32_H_37_NO_4_, the central di­hydro­pyridine ring adopts a flattened-boat conformation, with the N atom and the methine C atom displaced from the mean plane of the other four atoms by 0.0513 (14) and 0.1828 (18) Å, respectively. The two cyclo­hexenone rings adopt envelope conformations, with the tetra­subsituted C atoms as the flap atoms. The 3,4-di­meth­oxy­­benzene and benzyl rings are almost normal to the di­hydro­pyridine mean plane, with dihedral angles of 89.47 (9) and 82.90 (11)°, respectively. In the crystal, mol­ecules are linked *via* a pair of C—H⋯O hydrogen bonds, forming inversion dimers, which are, in turn, linked by C—H⋯O hydrogen bonds, forming slabs lying parallel to (001).

## Related literature   

For therapeutic properties of acridine derivatives, see: Nasim & Brychcy (1979 ▸); Thull & Testa (1994 ▸). For the crystal structures of similar deca­hydro­acridine-1,8-diones, see: Sughanya & Sureshbabu (2012 ▸); Abdelhamid *et al.* (2011 ▸); Akkurt *et al.* (2014 ▸); Khalilov *et al.* (2011 ▸); Tang *et al.* (2008 ▸); Tu *et al.* (2004 ▸). For a related synthesis, see: Li *et al.* (2003 ▸); Sughanya & Sureshbabu (2012 ▸). For ring conformation analysis, see: Cremer & Pople (1975 ▸).
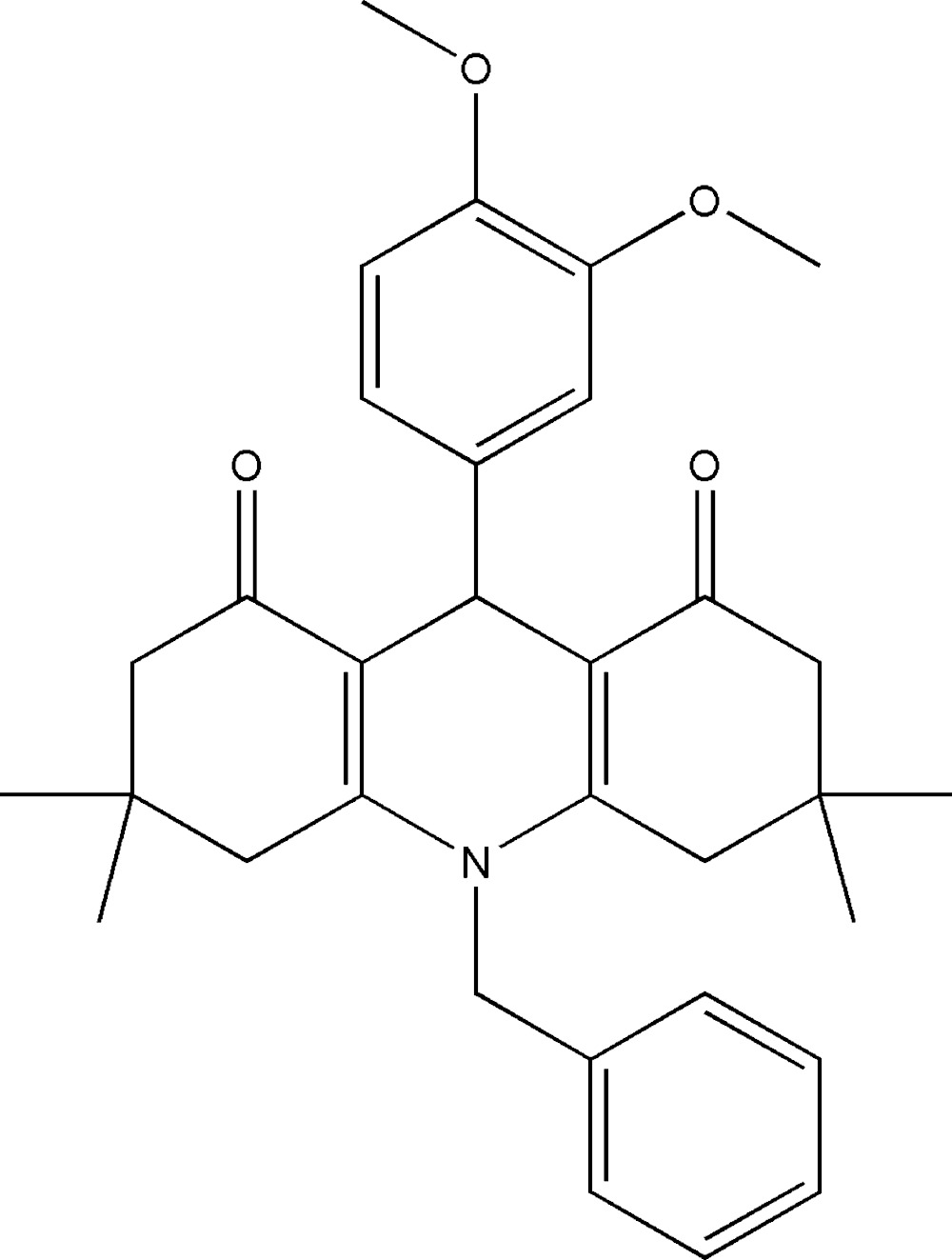


## Experimental   

### Crystal data  

C_32_H_37_NO_4_
*M* *_r_* = 499.63
Orthorhombic, 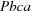

*a* = 10.7068 (3) Å
*b* = 17.8750 (4) Å
*c* = 28.1694 (7) Å
*V* = 5391.2 (2) Å^3^
*Z* = 8
Mo *K*α radiation
μ = 0.08 mm^−1^
*T* = 296 K
0.35 × 0.35 × 0.30 mm

### Data collection  

Bruker Kappa APEXII CCD diffractometer
Absorption correction: multi-scan (*SADABS*; Bruker, 2004 ▸) *T*_min_ = 0.958, *T*_max_ = 0.989
23489 measured reflections
4661 independent reflections
2966 reflections with *I* > 2σ(*I*)
*R*_int_ = 0.045

### Refinement  

*R*[*F* ^2^ > 2σ(*F* ^2^)] = 0.042
*wR*(*F* ^2^) = 0.119
*S* = 1.01
4661 reflections
335 parameters
H-atom parameters constrained
Δρ_max_ = 0.14 e Å^−3^
Δρ_min_ = −0.14 e Å^−3^

### 

Data collection: *APEX2* (Bruker, 2004 ▸); cell refinement: *APEX2* and *SAINT* (Bruker, 2004 ▸); data reduction: *SAINT* and *XPREP* (Bruker, 2004 ▸); program(s) used to solve structure: *SIR92* (Altomare *et al.*, 1993 ▸); program(s) used to refine structure: *SHELXL97* (Sheldrick, 2008 ▸); molecular graphics: *ORTEP-3 for Windows* (Farrugia, 2012 ▸) and *Mercury* (Macrae *et al.*, 2008 ▸); software used to prepare material for publication: *SHELXL97* (Sheldrick, 2008 ▸).

## Supplementary Material

Crystal structure: contains datablock(s) global, I. DOI: 10.1107/S2056989015014966/su5190sup1.cif

Structure factors: contains datablock(s) I. DOI: 10.1107/S2056989015014966/su5190Isup2.hkl

Supporting information file. DOI: 10.1107/S2056989015014966/su5190Isup3.cml

**Fig. 1.**
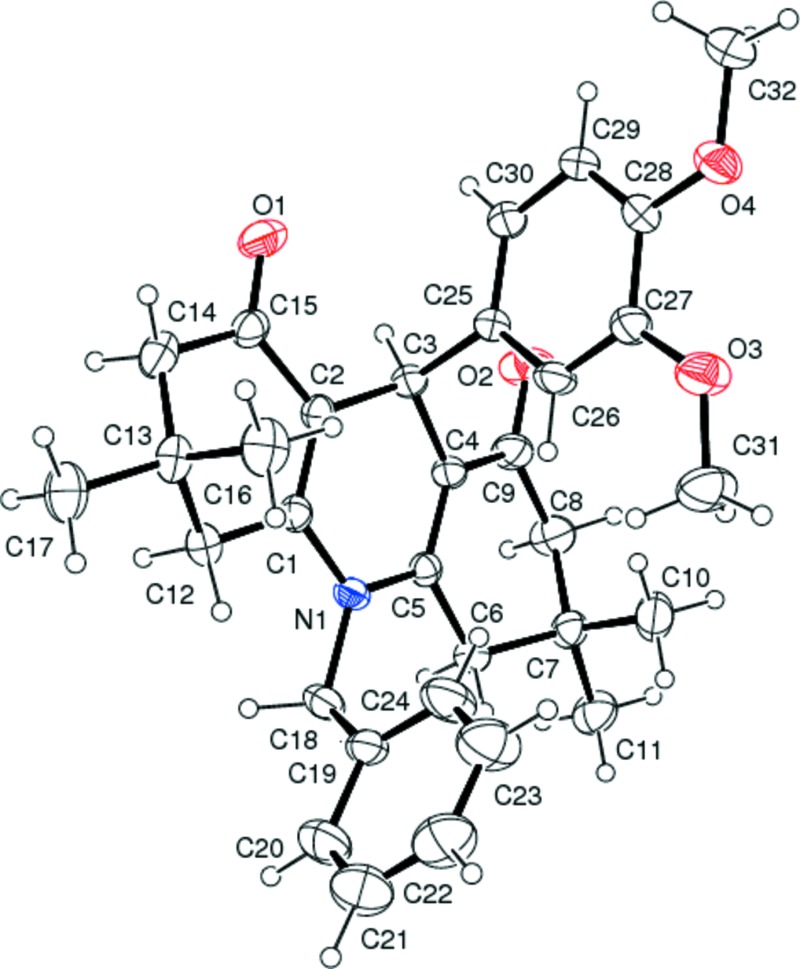
A view of the molecular structure of the title compound, with atom labelling. Displacement ellipsoids are drawn at the 30% probability level.

**Fig. 2.**
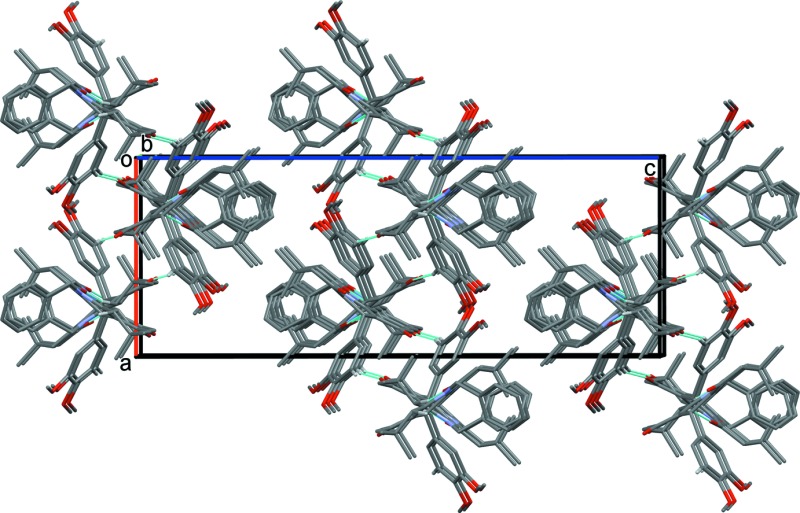
A view along the b axis of the crystal packing of the title compound. The C—H···O hydrogen bonds are shown as dashed lines (see Table 1).

. DOI: 10.1107/S2056989015014966/su5190fig1.tif
A view of the mol­ecular structure of the title compound, with atom labelling. Displacement ellipsoids are drawn at the 30% probability level.

b . DOI: 10.1107/S2056989015014966/su5190fig2.tif
A view along the *b* axis of the crystal packing of the title compound. The C—H⋯O hydrogen bonds are shown as dashed lines (see Table 1).

CCDC reference: 1417923

Additional supporting information:  crystallographic information; 3D view; checkCIF report

## Figures and Tables

**Table 1:** Hydrogen-bond geometry (, )

*D*H*A*	*D*H	H*A*	*D* *A*	*D*H*A*
C29H29O2^i^	0.93	2.39	3.293(3)	165
C6H6*B*O1^ii^	0.97	2.40	3.292(2)	154

Symmetry codes: (i) 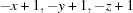; (ii) 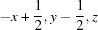.

## supplementary crystallographic information

### S1. Comment

\ Acridine derivatives with a di­hydro­pyridine unit belong to a special class of compounds, which are important because of their wide range of applications in the pharmaceutical and dye industries. They are also well known as therapeutic agents (Nasim & Brychcy, 1979; Thull & Testa, 1994).

In the title compound, Fig. 1, the bond lengths are close to those reported for similar compounds, for example 10-benzyl-9-(4-eth­oxy­phenyl)-3,3,6,6-tetra­methyl-3,4,6,7,9,10-\ hexa­hydro­acridine-1,8(2*H*,5*H*)-dione (Sughanya & Sureshbabu, 2012). In the di­hydro­pyridine ring bonds C4–C5 and C1–C2 are double bonds as indicated by the bond distances (C4–C5 = 1.349 (2) Å and C1–C2 = 1.348 (2) Å), . The C5–C4–C9 [119.84 (18)°] and C1–C2–C15 [120.15 (17)°] angles are almost the same. The central di­hydro­pyridine ring is almost planar with a mean deviation from the mean plane of 0.0509 (6) Å and with a maximum deviation of 0.0973 (3) Å for atom C3. The planar 3,4-di­meth­oxy­phenyl and benzyl rings form dihedral angles of 89.47 (9)° and 82.90 (11)° with the di­hydro­pyridine mean plane. Rings A (C4–C9), B (N1/C1—C5) and C (C1/C2/C12—C15) show total puckering amplitudes Q(T) of 0.469 (2) Å, 0.142 (1) Å and 0.484 (3) Å, respectively. The cyclo­hexenone rings A and C adopt envelope conformations, whereas the central ring B adopts a flattened boat conformation. This can be understood from the puckering parameters (Cremer & Pople, 1975): φ = 185.73 (2)° and θ = 58.67 (2)° (for A); φ = 0.3 (4)°, and θ = 111.1 (3)° (for B) and φ = 62.69 (2)°, θ = 120.98 (2)° (for C), respectively. In this conformation atoms C7 and C13 must be described as the flap atoms, being situated out of the plane of the respective rings with deviations of 0.3302 (2) Å and 0.3411 Å, respectively.

In the crystal, molecules are linked *via* a pair of C—H···O hydrogen bonds forming inversion dimers, which in turn are linked by C—H···O hydrogen bonds forming slabs lying parallel to (001); see Table 1 and Fig. 2

### S2. Synthesis and crystallization

The title compound was prepared in two stages. In the first stage, a mixture of 3,4-di­meth­oxy­benzaldehyde (0.83 g, 5 mmol), 5,5-di­methyl­cyclo­hexane-1,3-dione (dimedone) (1.40 g, 10 mmol) and 20 ml of ethanol was heated to 343 K for *ca* 10 min. The reaction mixture was allowed to cool to room temperature and the resulting solid inter­mediate, 2,2'-((3,4- di­meth­oxy­phenyl) methyl­ene)bis­(3-hy­droxy-5,5-di­methyl­cyclo­hex-2-enone) was filtered and dried (m.p.: 411 - 413 K; yield: 96%). In the second stage, *ca* 1.0 g (2.4 mmol) of this inter­mediate was dissolved in 25 ml of acetic acid. The solution was refluxed together with benzyl­amine (0.33 g, 3 mmol) for 8 h with the reaction being monitored by TLC. After completion of the reaction, the reaction mixture was poured into crushed ice and stirred well. The solid that separated was filtered and dried and then recrystallized from ethanol to yield yellow crystals of the title compound (m.p.: 449 - 451 K; yield: 76%).

### S3. Refinement

Crystal data, data collection and structure refinement details are summarized in Table 2. All the H atoms were identified from difference electron density maps and subsequently treated as riding atoms: C—H = 0.93 - 0.98 Å with U_iso_(H) = 1.5U_eq_(C) for methyl H atoms and 1.2U_eq_(C) for other H atoms.

### Crystal data

**Table:**

C_32_H_37_NO_4_	*D*_x_ = 1.231 Mg m^−^^3^
*M**_r_* = 499.63	Melting point = 449–451 K
Orthorhombic, *P**b**c**a*	Mo *K*α radiation, λ = 0.71073 Å
Hall symbol: -P 2a c2 ab	Cell parameters from 3680 reflections
*a* = 10.7068 (3) Å	θ = 2.3–23.8°
*b* = 17.8750 (4) Å	µ = 0.08 mm^−^^1^
*c* = 28.1694 (7) Å	*T* = 296 K
*V* = 5391.2 (2) Å^3^	Block, yellow
*Z* = 8	0.35 × 0.35 × 0.30 mm
*F*(000) = 2144	

### Data collection

**Table:**

Bruker Kappa APEXII CCD diffractometer	4661 independent reflections
Radiation source: fine-focus sealed tube	2966 reflections with *I* > 2σ(*I*)
Graphite monochromator	*R*_int_ = 0.045
ω and φ scan	θ_max_ = 24.9°, θ_min_ = 2.3°
Absorption correction: multi-scan (*SADABS*; Bruker, 2004)	*h* = −12→12
*T*_min_ = 0.958, *T*_max_ = 0.989	*k* = −20→20
23489 measured reflections	*l* = −33→27

### Refinement

**Table:**

Refinement on *F*^2^	Secondary atom site location: difference Fourier map
Least-squares matrix: full	Hydrogen site location: inferred from neighbouring sites
*R*[*F*^2^ > 2σ(*F*^2^)] = 0.042	H-atom parameters constrained
*wR*(*F*^2^) = 0.119	*w* = 1/[σ^2^(*F*_o_^2^) + (0.0522*P*)^2^ + 0.9815*P*] where *P* = (*F*_o_^2^ + 2*F*_c_^2^)/3
*S* = 1.01	(Δ/σ)_max_ = 0.002
4661 reflections	Δρ_max_ = 0.14 e Å^−^^3^
335 parameters	Δρ_min_ = −0.14 e Å^−^^3^
0 restraints	Extinction correction: *SHELXL97* (Sheldrick, 2008), Fc^*^=kFc[1+0.001xFc^2^λ^3^/sin(2θ)]^-1/4^
Primary atom site location: structure-invariant direct methods	Extinction coefficient: 0.0024 (3)

### Special details

**Table:**

Geometry. All e.s.d.'s (except the e.s.d. in the dihedral angle between two l.s. planes) are estimated using the full covariance matrix. The cell e.s.d.'s are taken into account individually in the estimation of e.s.d.'s in distances, angles and torsion angles; correlations between e.s.d.'s in cell parameters are only used when they are defined by crystal symmetry. An approximate (isotropic) treatment of cell e.s.d.'s is used for estimating e.s.d.'s involving l.s. planes.
Refinement. Refinement of *F*^2^ against ALL reflections. The weighted *R*-factor *wR* and goodness of fit *S* are based on *F*^2^, conventional *R*-factors *R* are based on *F*, with *F* set to zero for negative *F*^2^. The threshold expression of *F*^2^ > σ(*F*^2^) is used only for calculating *R*-factors(gt) *etc*. and is not relevant to the choice of reflections for refinement. *R*-factors based on *F*^2^ are statistically about twice as large as those based on *F*, and *R*- factors based on ALL data will be even larger.

### Fractional atomic coordinates and isotropic or equivalent isotropic displacement parameters (Å^2^)

**Table:**

	*x*	*y*	*z*	*U*_iso_*/*U*_eq_	
C1	0.14368 (15)	0.30051 (11)	0.61594 (6)	0.0354 (5)	
C2	0.18793 (16)	0.36442 (10)	0.59693 (6)	0.0357 (5)	
C3	0.28362 (16)	0.36551 (10)	0.55767 (7)	0.0376 (5)	
H3	0.2530	0.3996	0.5330	0.045*	
C4	0.29413 (16)	0.28887 (10)	0.53588 (6)	0.0354 (5)	
C5	0.24760 (15)	0.22657 (10)	0.55628 (6)	0.0332 (4)	
C6	0.26823 (17)	0.15026 (11)	0.53557 (7)	0.0404 (5)	
H6A	0.1936	0.1354	0.5185	0.048*	
H6B	0.2805	0.1149	0.5613	0.048*	
C7	0.37920 (17)	0.14543 (11)	0.50209 (7)	0.0436 (5)	
C8	0.3691 (2)	0.20922 (12)	0.46697 (8)	0.0563 (6)	
H8A	0.4436	0.2098	0.4473	0.068*	
H8B	0.2982	0.2002	0.4463	0.068*	
C9	0.35425 (19)	0.28421 (12)	0.48963 (7)	0.0478 (5)	
C10	0.50057 (19)	0.15092 (14)	0.53043 (9)	0.0641 (7)	
H10A	0.5048	0.1104	0.5527	0.096*	
H10B	0.5027	0.1976	0.5472	0.096*	
H10C	0.5705	0.1482	0.5092	0.096*	
C11	0.3746 (2)	0.07038 (13)	0.47679 (8)	0.0628 (7)	
H11A	0.3813	0.0308	0.4997	0.094*	
H11B	0.4427	0.0671	0.4547	0.094*	
H11C	0.2969	0.0659	0.4600	0.094*	
C12	0.05426 (17)	0.30154 (11)	0.65704 (7)	0.0442 (5)	
H12A	0.0690	0.2577	0.6766	0.053*	
H12B	−0.0304	0.2984	0.6449	0.053*	
C13	0.06601 (17)	0.37125 (12)	0.68782 (7)	0.0465 (5)	
C14	0.05534 (19)	0.43847 (12)	0.65534 (7)	0.0523 (6)	
H14A	−0.0299	0.4418	0.6439	0.063*	
H14B	0.0724	0.4833	0.6736	0.063*	
C15	0.14163 (17)	0.43654 (12)	0.61355 (7)	0.0428 (5)	
C16	0.1906 (2)	0.37117 (14)	0.71379 (8)	0.0638 (7)	
H16A	0.1968	0.4152	0.7331	0.096*	
H16B	0.2574	0.3707	0.6911	0.096*	
H16C	0.1961	0.3275	0.7335	0.096*	
C17	−0.0396 (2)	0.37115 (15)	0.72388 (8)	0.0689 (7)	
H17A	−0.0336	0.4149	0.7435	0.103*	
H17B	−0.0336	0.3272	0.7434	0.103*	
H17C	−0.1183	0.3712	0.7076	0.103*	
C18	0.12476 (18)	0.16313 (11)	0.61951 (7)	0.0467 (5)	
H18A	0.1285	0.1225	0.5967	0.056*	
H18B	0.0375	0.1718	0.6270	0.056*	
C19	0.19151 (19)	0.13942 (11)	0.66410 (7)	0.0464 (5)	
C20	0.1352 (2)	0.08931 (14)	0.69419 (9)	0.0724 (7)	
H20	0.0572	0.0699	0.6864	0.087*	
C21	0.1919 (3)	0.06730 (17)	0.73565 (11)	0.0921 (9)	
H21	0.1520	0.0334	0.7556	0.111*	
C22	0.3054 (3)	0.09451 (16)	0.74766 (10)	0.0839 (8)	
H22	0.3432	0.0801	0.7759	0.101*	
C23	0.3631 (3)	0.14294 (16)	0.71809 (10)	0.0874 (9)	
H23	0.4418	0.1614	0.7258	0.105*	
C24	0.3064 (2)	0.16514 (14)	0.67670 (9)	0.0708 (7)	
H24	0.3476	0.1985	0.6568	0.085*	
C25	0.40789 (16)	0.39613 (10)	0.57537 (7)	0.0377 (5)	
C26	0.48182 (18)	0.35503 (11)	0.60659 (7)	0.0446 (5)	
H26	0.4570	0.3071	0.6153	0.054*	
C27	0.59087 (18)	0.38395 (12)	0.62477 (7)	0.0467 (5)	
C28	0.62704 (17)	0.45648 (11)	0.61294 (7)	0.0446 (5)	
C29	0.55694 (18)	0.49609 (11)	0.58102 (8)	0.0484 (5)	
H29	0.5821	0.5437	0.5718	0.058*	
C30	0.44861 (18)	0.46556 (11)	0.56234 (7)	0.0463 (5)	
H30	0.4026	0.4930	0.5404	0.056*	
C31	0.6482 (2)	0.26982 (14)	0.66202 (10)	0.0810 (8)	
H31A	0.7101	0.2501	0.6833	0.121*	
H31B	0.6522	0.2437	0.6323	0.121*	
H31C	0.5668	0.2634	0.6757	0.121*	
C32	0.7733 (2)	0.55530 (13)	0.62149 (9)	0.0683 (7)	
H32A	0.8487	0.5674	0.6382	0.102*	
H32B	0.7093	0.5906	0.6297	0.102*	
H32C	0.7884	0.5574	0.5879	0.102*	
N1	0.17583 (13)	0.23059 (8)	0.59747 (5)	0.0367 (4)	
O1	0.16937 (14)	0.49427 (8)	0.59302 (6)	0.0620 (4)	
O2	0.38837 (17)	0.34063 (9)	0.46896 (6)	0.0783 (5)	
O3	0.67062 (14)	0.34610 (9)	0.65443 (6)	0.0707 (5)	
O4	0.73418 (13)	0.48237 (8)	0.63413 (5)	0.0607 (4)	

### Atomic displacement parameters (Å^2^)

**Table:**

	*U*^11^	*U*^22^	*U*^33^	*U*^12^	*U*^13^	*U*^23^
C1	0.0291 (9)	0.0384 (12)	0.0388 (11)	0.0007 (8)	−0.0010 (8)	−0.0018 (9)
C2	0.0320 (9)	0.0352 (12)	0.0401 (11)	0.0017 (8)	−0.0044 (8)	0.0013 (9)
C3	0.0385 (10)	0.0331 (11)	0.0411 (11)	0.0010 (8)	−0.0008 (8)	0.0075 (9)
C4	0.0362 (10)	0.0337 (12)	0.0363 (11)	−0.0002 (8)	0.0001 (8)	0.0058 (9)
C5	0.0314 (9)	0.0356 (11)	0.0326 (10)	0.0012 (8)	−0.0012 (8)	0.0016 (9)
C6	0.0412 (10)	0.0377 (12)	0.0423 (11)	−0.0003 (9)	0.0003 (9)	0.0022 (9)
C7	0.0463 (11)	0.0430 (13)	0.0416 (12)	0.0050 (9)	0.0058 (9)	−0.0002 (10)
C8	0.0738 (15)	0.0527 (15)	0.0423 (13)	0.0035 (11)	0.0132 (11)	0.0024 (11)
C9	0.0567 (12)	0.0444 (13)	0.0424 (13)	0.0012 (10)	0.0068 (10)	0.0110 (11)
C10	0.0453 (12)	0.0692 (17)	0.0777 (17)	0.0092 (11)	0.0012 (12)	−0.0047 (13)
C11	0.0737 (16)	0.0539 (15)	0.0607 (15)	0.0107 (12)	0.0109 (12)	−0.0092 (12)
C12	0.0354 (10)	0.0514 (14)	0.0459 (12)	0.0003 (9)	0.0040 (9)	0.0001 (10)
C13	0.0384 (11)	0.0563 (14)	0.0449 (12)	0.0082 (10)	0.0009 (9)	−0.0064 (11)
C14	0.0471 (12)	0.0513 (14)	0.0586 (14)	0.0118 (10)	−0.0035 (10)	−0.0107 (11)
C15	0.0381 (11)	0.0385 (13)	0.0518 (13)	0.0045 (9)	−0.0090 (9)	−0.0009 (11)
C16	0.0562 (13)	0.0824 (18)	0.0527 (14)	0.0055 (12)	−0.0120 (11)	−0.0073 (13)
C17	0.0598 (14)	0.0882 (19)	0.0586 (15)	0.0104 (13)	0.0165 (12)	−0.0123 (13)
C18	0.0490 (11)	0.0409 (13)	0.0501 (13)	−0.0135 (9)	0.0134 (10)	−0.0001 (10)
C19	0.0533 (12)	0.0352 (12)	0.0505 (13)	−0.0025 (10)	0.0139 (10)	0.0038 (10)
C20	0.0720 (16)	0.0712 (18)	0.0739 (18)	−0.0128 (13)	0.0129 (14)	0.0256 (15)
C21	0.104 (2)	0.092 (2)	0.081 (2)	−0.0020 (19)	0.0204 (18)	0.0411 (17)
C22	0.104 (2)	0.081 (2)	0.0658 (18)	0.0075 (18)	−0.0068 (17)	0.0229 (16)
C23	0.091 (2)	0.084 (2)	0.087 (2)	−0.0144 (16)	−0.0244 (17)	0.0286 (18)
C24	0.0705 (16)	0.0709 (18)	0.0711 (17)	−0.0179 (13)	−0.0053 (14)	0.0282 (14)
C25	0.0371 (10)	0.0325 (11)	0.0434 (11)	−0.0015 (8)	0.0034 (9)	0.0048 (9)
C26	0.0468 (12)	0.0356 (12)	0.0515 (13)	−0.0091 (9)	−0.0009 (10)	0.0125 (10)
C27	0.0438 (11)	0.0437 (13)	0.0525 (13)	−0.0022 (10)	−0.0051 (10)	0.0100 (10)
C28	0.0363 (11)	0.0419 (13)	0.0557 (13)	−0.0057 (9)	0.0024 (9)	0.0009 (11)
C29	0.0431 (11)	0.0326 (12)	0.0694 (14)	−0.0042 (9)	0.0043 (11)	0.0075 (11)
C30	0.0425 (11)	0.0403 (13)	0.0560 (13)	0.0010 (9)	0.0001 (10)	0.0108 (10)
C31	0.0877 (19)	0.0586 (18)	0.097 (2)	−0.0042 (14)	−0.0341 (16)	0.0275 (15)
C32	0.0583 (14)	0.0491 (15)	0.0973 (19)	−0.0173 (12)	−0.0052 (13)	−0.0046 (14)
N1	0.0393 (8)	0.0320 (9)	0.0389 (9)	−0.0048 (7)	0.0072 (7)	0.0022 (7)
O1	0.0742 (11)	0.0371 (9)	0.0748 (11)	0.0078 (8)	0.0049 (8)	0.0051 (8)
O2	0.1186 (14)	0.0546 (11)	0.0617 (11)	−0.0040 (10)	0.0373 (10)	0.0168 (9)
O3	0.0679 (10)	0.0570 (11)	0.0873 (12)	−0.0124 (8)	−0.0327 (9)	0.0247 (9)
O4	0.0490 (8)	0.0525 (10)	0.0807 (11)	−0.0145 (7)	−0.0122 (8)	0.0048 (8)

### Geometric parameters (Å, º)

**Table:**

C1—C2	1.348 (2)	C16—H16B	0.9600
C1—N1	1.397 (2)	C16—H16C	0.9600
C1—C12	1.503 (3)	C17—H17A	0.9600
C2—C15	1.458 (3)	C17—H17B	0.9600
C2—C3	1.508 (2)	C17—H17C	0.9600
C3—C4	1.505 (3)	C18—N1	1.462 (2)
C3—C25	1.523 (2)	C18—C19	1.506 (3)
C3—H3	0.9800	C18—H18A	0.9700
C4—C5	1.349 (2)	C18—H18B	0.9700
C4—C9	1.456 (3)	C19—C24	1.361 (3)
C5—N1	1.393 (2)	C19—C20	1.373 (3)
C5—C6	1.500 (3)	C20—C21	1.374 (4)
C6—C7	1.519 (3)	C20—H20	0.9300
C6—H6A	0.9700	C21—C22	1.352 (4)
C6—H6B	0.9700	C21—H21	0.9300
C7—C8	1.513 (3)	C22—C23	1.351 (4)
C7—C11	1.520 (3)	C22—H22	0.9300
C7—C10	1.528 (3)	C23—C24	1.373 (3)
C8—C9	1.493 (3)	C23—H23	0.9300
C8—H8A	0.9700	C24—H24	0.9300
C8—H8B	0.9700	C25—C30	1.366 (3)
C9—O2	1.220 (2)	C25—C26	1.393 (3)
C10—H10A	0.9600	C26—C27	1.376 (3)
C10—H10B	0.9600	C26—H26	0.9300
C10—H10C	0.9600	C27—O3	1.373 (2)
C11—H11A	0.9600	C27—C28	1.393 (3)
C11—H11B	0.9600	C28—C29	1.369 (3)
C11—H11C	0.9600	C28—O4	1.373 (2)
C12—C13	1.523 (3)	C29—C30	1.386 (3)
C12—H12A	0.9700	C29—H29	0.9300
C12—H12B	0.9700	C30—H30	0.9300
C13—C14	1.515 (3)	C31—O3	1.401 (3)
C13—C17	1.520 (3)	C31—H31A	0.9600
C13—C16	1.521 (3)	C31—H31B	0.9600
C14—C15	1.497 (3)	C31—H31C	0.9600
C14—H14A	0.9700	C32—O4	1.415 (3)
C14—H14B	0.9700	C32—H32A	0.9600
C15—O1	1.220 (2)	C32—H32B	0.9600
C16—H16A	0.9600	C32—H32C	0.9600
			
C2—C1—N1	121.59 (16)	C13—C16—H16A	109.5
C2—C1—C12	121.32 (17)	C13—C16—H16B	109.5
N1—C1—C12	117.06 (16)	H16A—C16—H16B	109.5
C1—C2—C15	120.15 (17)	C13—C16—H16C	109.5
C1—C2—C3	122.78 (17)	H16A—C16—H16C	109.5
C15—C2—C3	117.06 (16)	H16B—C16—H16C	109.5
C4—C3—C2	109.76 (15)	C13—C17—H17A	109.5
C4—C3—C25	113.28 (15)	C13—C17—H17B	109.5
C2—C3—C25	110.99 (15)	H17A—C17—H17B	109.5
C4—C3—H3	107.5	C13—C17—H17C	109.5
C2—C3—H3	107.5	H17A—C17—H17C	109.5
C25—C3—H3	107.5	H17B—C17—H17C	109.5
C5—C4—C9	119.84 (18)	N1—C18—C19	114.13 (16)
C5—C4—C3	123.38 (16)	N1—C18—H18A	108.7
C9—C4—C3	116.74 (16)	C19—C18—H18A	108.7
C4—C5—N1	121.06 (17)	N1—C18—H18B	108.7
C4—C5—C6	122.06 (16)	C19—C18—H18B	108.7
N1—C5—C6	116.87 (15)	H18A—C18—H18B	107.6
C5—C6—C7	114.09 (16)	C24—C19—C20	117.2 (2)
C5—C6—H6A	108.7	C24—C19—C18	123.47 (19)
C7—C6—H6A	108.7	C20—C19—C18	119.3 (2)
C5—C6—H6B	108.7	C19—C20—C21	121.1 (3)
C7—C6—H6B	108.7	C19—C20—H20	119.4
H6A—C6—H6B	107.6	C21—C20—H20	119.4
C8—C7—C6	107.90 (16)	C22—C21—C20	120.5 (3)
C8—C7—C11	110.87 (17)	C22—C21—H21	119.8
C6—C7—C11	108.41 (16)	C20—C21—H21	119.8
C8—C7—C10	110.71 (18)	C23—C22—C21	119.2 (3)
C6—C7—C10	109.70 (16)	C23—C22—H22	120.4
C11—C7—C10	109.21 (17)	C21—C22—H22	120.4
C9—C8—C7	113.86 (17)	C22—C23—C24	120.4 (3)
C9—C8—H8A	108.8	C22—C23—H23	119.8
C7—C8—H8A	108.8	C24—C23—H23	119.8
C9—C8—H8B	108.8	C19—C24—C23	121.6 (2)
C7—C8—H8B	108.8	C19—C24—H24	119.2
H8A—C8—H8B	107.7	C23—C24—H24	119.2
O2—C9—C4	120.81 (19)	C30—C25—C26	117.88 (17)
O2—C9—C8	120.39 (19)	C30—C25—C3	121.17 (17)
C4—C9—C8	118.76 (18)	C26—C25—C3	120.92 (16)
C7—C10—H10A	109.5	C27—C26—C25	121.28 (18)
C7—C10—H10B	109.5	C27—C26—H26	119.4
H10A—C10—H10B	109.5	C25—C26—H26	119.4
C7—C10—H10C	109.5	O3—C27—C26	124.69 (18)
H10A—C10—H10C	109.5	O3—C27—C28	115.54 (17)
H10B—C10—H10C	109.5	C26—C27—C28	119.76 (18)
C7—C11—H11A	109.5	C29—C28—O4	124.68 (18)
C7—C11—H11B	109.5	C29—C28—C27	119.09 (18)
H11A—C11—H11B	109.5	O4—C28—C27	116.22 (18)
C7—C11—H11C	109.5	C28—C29—C30	120.31 (19)
H11A—C11—H11C	109.5	C28—C29—H29	119.8
H11B—C11—H11C	109.5	C30—C29—H29	119.8
C1—C12—C13	113.33 (16)	C25—C30—C29	121.54 (19)
C1—C12—H12A	108.9	C25—C30—H30	119.2
C13—C12—H12A	108.9	C29—C30—H30	119.2
C1—C12—H12B	108.9	O3—C31—H31A	109.5
C13—C12—H12B	108.9	O3—C31—H31B	109.5
H12A—C12—H12B	107.7	H31A—C31—H31B	109.5
C14—C13—C17	110.40 (17)	O3—C31—H31C	109.5
C14—C13—C16	110.94 (18)	H31A—C31—H31C	109.5
C17—C13—C16	109.33 (17)	H31B—C31—H31C	109.5
C14—C13—C12	107.39 (16)	O4—C32—H32A	109.5
C17—C13—C12	108.54 (17)	O4—C32—H32B	109.5
C16—C13—C12	110.20 (17)	H32A—C32—H32B	109.5
C15—C14—C13	114.23 (16)	O4—C32—H32C	109.5
C15—C14—H14A	108.7	H32A—C32—H32C	109.5
C13—C14—H14A	108.7	H32B—C32—H32C	109.5
C15—C14—H14B	108.7	C5—N1—C1	119.48 (15)
C13—C14—H14B	108.7	C5—N1—C18	121.14 (15)
H14A—C14—H14B	107.6	C1—N1—C18	119.18 (15)
O1—C15—C2	120.86 (18)	C27—O3—C31	117.76 (17)
O1—C15—C14	120.25 (18)	C28—O4—C32	116.64 (17)
C2—C15—C14	118.85 (18)		
			
N1—C1—C2—C15	173.04 (16)	C13—C14—C15—C2	23.8 (3)
C12—C1—C2—C15	−5.0 (3)	N1—C18—C19—C24	−16.1 (3)
N1—C1—C2—C3	−5.7 (3)	N1—C18—C19—C20	163.84 (19)
C12—C1—C2—C3	176.20 (16)	C24—C19—C20—C21	1.1 (4)
C1—C2—C3—C4	14.5 (2)	C18—C19—C20—C21	−178.8 (2)
C15—C2—C3—C4	−164.33 (15)	C19—C20—C21—C22	−0.3 (4)
C1—C2—C3—C25	−111.51 (19)	C20—C21—C22—C23	−0.8 (5)
C15—C2—C3—C25	69.7 (2)	C21—C22—C23—C24	1.0 (5)
C2—C3—C4—C5	−14.7 (2)	C20—C19—C24—C23	−1.0 (4)
C25—C3—C4—C5	110.01 (19)	C18—C19—C24—C23	178.9 (2)
C2—C3—C4—C9	163.11 (16)	C22—C23—C24—C19	−0.1 (4)
C25—C3—C4—C9	−72.2 (2)	C4—C3—C25—C30	128.47 (19)
C9—C4—C5—N1	−171.74 (16)	C2—C3—C25—C30	−107.5 (2)
C3—C4—C5—N1	6.0 (3)	C4—C3—C25—C26	−53.5 (2)
C9—C4—C5—C6	7.1 (3)	C2—C3—C25—C26	70.5 (2)
C3—C4—C5—C6	−175.15 (16)	C30—C25—C26—C27	1.6 (3)
C4—C5—C6—C7	20.8 (2)	C3—C25—C26—C27	−176.42 (18)
N1—C5—C6—C7	−160.26 (15)	C25—C26—C27—O3	−177.78 (19)
C5—C6—C7—C8	−49.3 (2)	C25—C26—C27—C28	1.8 (3)
C5—C6—C7—C11	−169.41 (16)	O3—C27—C28—C29	175.64 (18)
C5—C6—C7—C10	71.4 (2)	C26—C27—C28—C29	−4.0 (3)
C6—C7—C8—C9	53.0 (2)	O3—C27—C28—O4	−3.1 (3)
C11—C7—C8—C9	171.55 (18)	C26—C27—C28—O4	177.31 (18)
C10—C7—C8—C9	−67.1 (2)	O4—C28—C29—C30	−178.64 (19)
C5—C4—C9—O2	174.36 (19)	C27—C28—C29—C30	2.7 (3)
C3—C4—C9—O2	−3.5 (3)	C26—C25—C30—C29	−2.9 (3)
C5—C4—C9—C8	−3.3 (3)	C3—C25—C30—C29	175.15 (18)
C3—C4—C9—C8	178.83 (17)	C28—C29—C30—C25	0.7 (3)
C7—C8—C9—O2	153.9 (2)	C4—C5—N1—C1	4.9 (2)
C7—C8—C9—C4	−28.4 (3)	C6—C5—N1—C1	−174.09 (15)
C2—C1—C12—C13	−26.5 (2)	C4—C5—N1—C18	179.68 (17)
N1—C1—C12—C13	155.38 (16)	C6—C5—N1—C18	0.7 (2)
C1—C12—C13—C14	53.1 (2)	C2—C1—N1—C5	−5.0 (3)
C1—C12—C13—C17	172.45 (17)	C12—C1—N1—C5	173.19 (15)
C1—C12—C13—C16	−67.9 (2)	C2—C1—N1—C18	−179.88 (17)
C17—C13—C14—C15	−169.96 (17)	C12—C1—N1—C18	−1.7 (2)
C16—C13—C14—C15	68.7 (2)	C19—C18—N1—C5	105.3 (2)
C12—C13—C14—C15	−51.8 (2)	C19—C18—N1—C1	−79.8 (2)
C1—C2—C15—O1	−171.21 (18)	C26—C27—O3—C31	8.5 (3)
C3—C2—C15—O1	7.6 (3)	C28—C27—O3—C31	−171.1 (2)
C1—C2—C15—C14	6.6 (3)	C29—C28—O4—C32	−0.1 (3)
C3—C2—C15—C14	−174.57 (16)	C27—C28—O4—C32	178.57 (19)
C13—C14—C15—O1	−158.43 (18)		

### Hydrogen-bond geometry (Å, º)

**Table:**

*D*—H···*A*	*D*—H	H···*A*	*D*···*A*	*D*—H···*A*
C29—H29···O2^i^	0.93	2.39	3.293 (3)	165
C6—H6*B*···O1^ii^	0.97	2.40	3.292 (2)	154

Symmetry codes: (i) −*x*+1, −*y*+1, −*z*+1; (ii) −*x*+1/2, *y*−1/2, *z*.

## References

[bb1] Abdelhamid, A. A., Mohamed, S. K., Khalilov, A. N., Gurbanov, A. V. & Ng, S. W. (2011). *Acta Cryst.* E**67**, o744. 10.1107/S1600536811006969 PMC3051986 21522483

[bb2] Akkurt, M., Mohamed, S. K., Abdelhamid, A. A., Gaber, A.-A. M. & Albayati, M. R. (2014). *Acta Cryst.* E**70**, o663–o664. 10.1107/S1600536814010460 PMC4051074 24940246

[bb3] Altomare, A., Cascarano, G., Giacovazzo, C. & Guagliardi, A. (1993). *J. Appl. Cryst.* **26**, 343–350.

[bb4] Bruker (2004). *APEX2*, *SAINT*, *XPREP* and *SADABS*. Bruker AXS Inc., Madison, Wisconsin, USA.

[bb5] Cremer, D. & Pople, J. A. (1975). *J. Am. Chem. Soc.* **97**, 1354–1358.

[bb6] Farrugia, L. J. (2012). *J. Appl. Cryst.* **45**, 849–854.

[bb7] Khalilov, A. N., Abdelhamid, A. A., Gurbanov, A. V. & Ng, S. W. (2011). *Acta Cryst.* E**67**, o1146. 10.1107/S1600536811013481 PMC3089333 21754454

[bb8] Li, Y., Wang, X., Shi, D., Du, B. & Tu, S. (2003). *Acta Cryst.* E**59**, o1446–o1448.

[bb9] Macrae, C. F., Bruno, I. J., Chisholm, J. A., Edgington, P. R., McCabe, P., Pidcock, E., Rodriguez-Monge, L., Taylor, R., van de Streek, J. & Wood, P. A. (2008). *J. Appl. Cryst.* **41**, 466–470.

[bb10] Nasim, A. & Brychcy, T. (1979). *Mutat. Res./Rev. Genet. Toxicol.* **65**, 261–288. 10.1016/0165-1110(79)90005-8 390382

[bb11] Sheldrick, G. M. (2008). *Acta Cryst.* A**64**, 112–122. 10.1107/S0108767307043930 18156677

[bb12] Sughanya, V. & Sureshbabu, N. (2012). *Acta Cryst.* E**68**, o2755. 10.1107/S1600536812036094 PMC3435766 22969637

[bb13] Tang, Z., Liu, C., Wu, S. & Hao, W. (2008). *Acta Cryst.* E**64**, o1844. 10.1107/S1600536808027256 PMC2960545 21201815

[bb14] Thull, U. & Testa, B. (1994). *Biochem. Pharmacol.* **47**, 2307–2310. 10.1016/0006-2952(94)90271-2 8031326

[bb15] Tu, S., Miao, C., Gao, Y., Fang, F., Zhuang, Q., Feng, Y. & Shi, D. (2004). *Synlett*, pp. 255–258.

