# Supplementary figures and images for: Crystal structure of 10-benzyl-9-(3,4-di­meth­oxy­phen­yl)-3,3,6,6-tetra­methyl-3,4,6,7,9,10-hexa­hydro­acridine-1,8(2H,5H)-dione
Source: Acta Crystallogr E Crystallogr Commun. 2015 Aug 26;71(Pt 9):o688–9. doi: 10.1107/S2056989015014966 (PMC4555413; doi:10.1107/S2056989015014966)

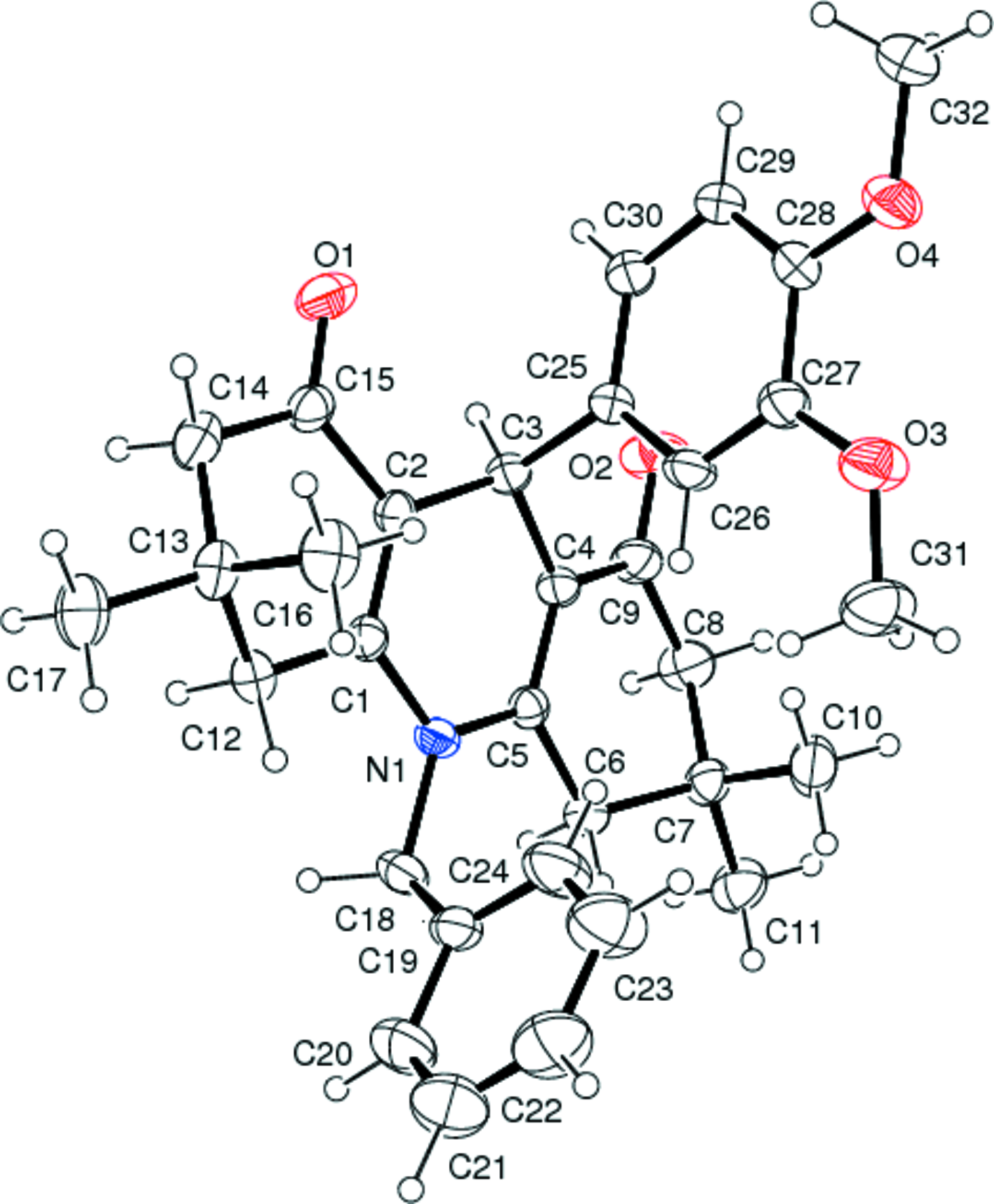

Supplement: Supplementary file 4 [file e-71-0o688-fig1.tif]

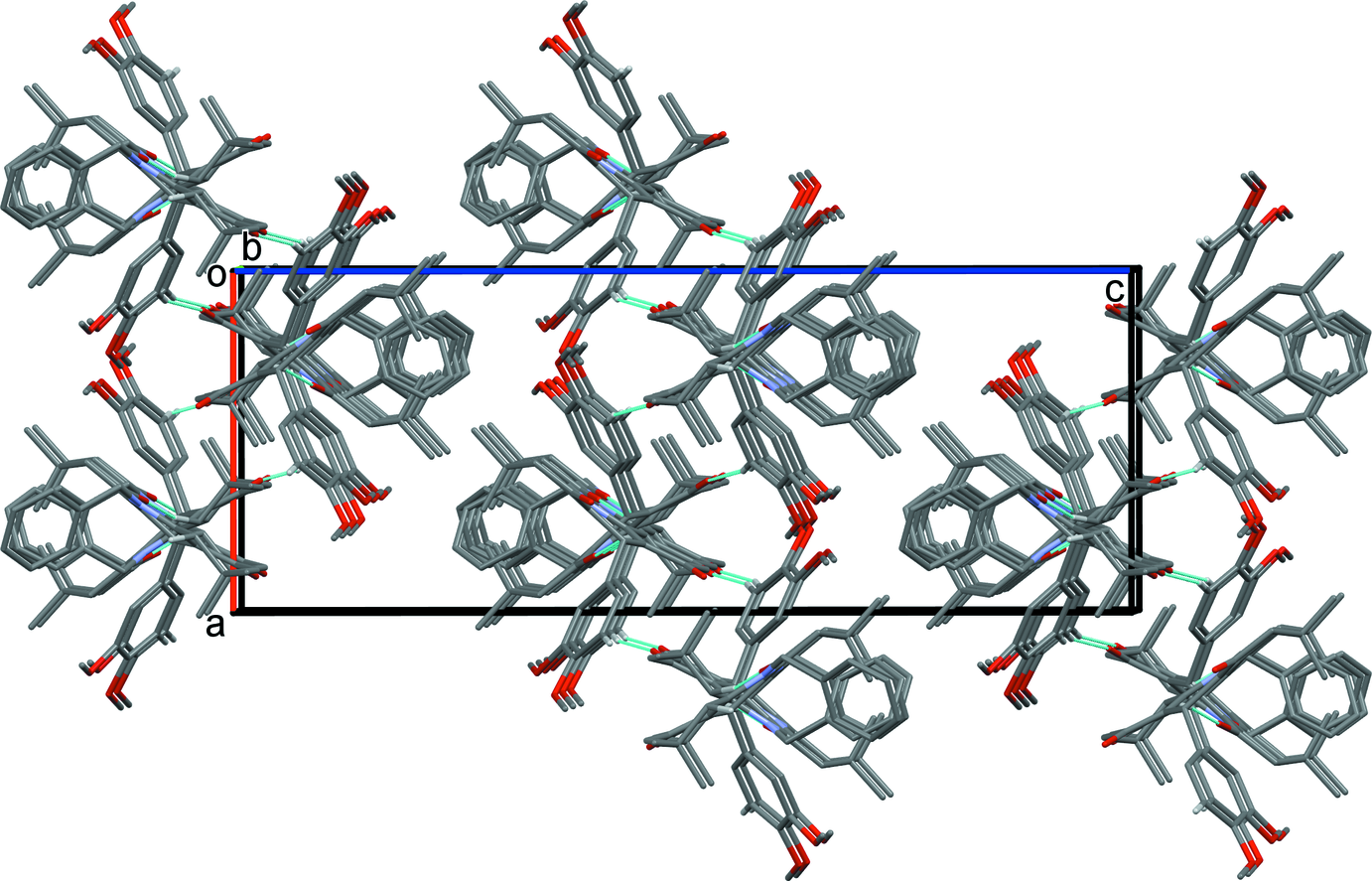

Supplement: Supplementary file 5 [file e-71-0o688-fig2.tif]
